# Common Biochemical and Magnetic Resonance Imaging Biomarkers of Early Knee Osteoarthritis and of Exercise/Training in Athletes: A Narrative Review

**DOI:** 10.3390/diagnostics11081488

**Published:** 2021-08-17

**Authors:** Johanne Martel-Pelletier, Ginette Tardif, Patrice Paiement, Jean-Pierre Pelletier

**Affiliations:** Osteoarthritis Research Unit, University of Montreal Hospital Research Centre (CRCHUM), Montreal, QC H2X 0A9, Canada; tardifgi@yahoo.com (G.T.); ppaiement@arthrolab.com (P.P.); dr@jppelletier.ca (J.-P.P.)

**Keywords:** early osteoarthritis, joint-loading sports, biomarkers, magnetic resonance imaging, athletes

## Abstract

Knee osteoarthritis (OA) is the most common joint disease of the world population. Although considered a disease of old age, OA also affects young individuals and, more specifically among them, those practicing knee-joint-loading sports. Predicting OA at an early stage is crucial but remains a challenge. Biomarkers that can predict early OA development will help in the design of specific therapeutic strategies for individuals and, for athletes, to avoid adverse outcomes due to exercising/training regimens. This review summarizes and compares the current knowledge of fluid and magnetic resonance imaging (MRI) biomarkers common to early knee OA and exercise/training in athletes. A variety of fluid biochemical markers have been proposed to detect knee OA at an early stage; however, few have shown similar behavior between the two studied groups. Moreover, in endurance athletes, they are often contingent on the sport involved. MRI has also demonstrated its ability for early detection of joint structural alterations in both groups. It is currently suggested that for optimal forecasting of early knee structural alterations, both fluid and MRI biomarkers should be analyzed as a panel and/or combined, rather than individually.

## 1. Introduction

Osteoarthritis (OA) is the most common joint disease worldwide. In recent years, this disease demonstrated an increase in both incidence and prevalence, affecting about 18% of the world population. This disease is a leading cause of global years lived with disability (YLDs) [1,2], and a great economic burden for society. The pathological processes culminate in pain, loss of joint function, and disability, and often require joint replacement [3].

Although the pathology of OA has long been thought to be cartilage-driven, it is now recognized that it is a complex disease affecting multiple tissues in the joint [4,5,6]. It can develop slowly or rapidly. The main risk factors for knee OA are age, gender, and body mass index (BMI) [7,8,9]. The disease’s current diagnostic approaches struggle with limited sensitivity and specificity, as well as prediction and monitoring of its progression. There is a real necessity for a reliable method for early OA detection, which would be a significant step toward OA precision medicine. Early OA detection would enable health care professionals to be more effective by allowing the adjustment of treatment plans and therapeutic approaches. It would ensure the right treatment at the right time, a key strategy for more effective therapies and results.

OA used to be considered a disease of old age and defined by radiographic changes observed therein and considered representative of the disease process. However, not only is the older population affected by this disease, but also young individuals and athletes. Age-dependent OA is the result of years or even decades of pathological processes preceding joint structural changes [10]. The initial events, which define early stage OA, consist of a prolonged period of molecular alterations detectable by fluid/serological analyses [11,12]. This stage of molecular changes is followed by articular tissue alterations. Early articular tissue damages detected by sensitive imaging techniques such as magnetic resonance imaging (MRI) [13,14,15] are also considered to be early stages of OA. This disease is generally diagnosed at a moderate stage of the disease, i.e., when articular pain occurs and/or articular structural damages become visible by radiography. This disease may also only be diagnosed at a severe stage; when articular pain is well established.

Exercise has long been advocated as beneficial in the management of OA [16,17,18,19,20]. However, there is a lack of clarity concerning the effects of exercise on knee joint structures. Our knowledge is perplexed by the contrasting effects of regular, moderate joint-loading exercise and those overloading the joints or being excessive. Some believe that sports with repetitive and excessive joint loading likely increase the risk of articular tissue degradation, resulting in the clinical symptoms of OA [7,21,22,23,24,25,26]. In contrast to age-dependent OA, the disease process occurs more rapidly in the athlete’s joint subjected to high mechanical stress and is associated with an accelerated progression [23,25,26,27,28,29,30]. For example, in soccer players, the incidence of OA is 5–12 times more frequent than in the general population and it is diagnosed 4–5 years earlier [31,32].

For both groups, more information on the evolution of OA is warranted in the search for targeted measures that will delay or stop its progression. To this end, OA biomarkers, molecules or markers involved in the early stages of the pathological OA process, could be used as warning signs, before serious joint damage occurs. This will permit health professionals to intervene with an appropriate action/therapeutic approach to reduce the likelihood of further knee structure alteration.

This review presents and compares fluid and MRI biomarkers common to early knee OA and endurance exercise/training athletes, to ascertain a common signature between these two pathological articular processes.

## 2. Biomarkers

Biomarkers generally stem from an awareness of the pathophysiology of a disease. They are invaluable tools in both predicting disease and discriminating pathological from physiological events. In brief, biomarkers offer an avenue of research to assess the early development of OA.

In this review, biomarkers are classified under two broad categories: biochemical molecules present in biological fluids, such as urine and blood/serum, and imaging markers from MRI. These were chosen as specific biological molecules could provide precise information on the metabolism of individuals at specific points in time. In addition to being a noninvasive tool, MRI provides complementary information on early structural alterations, not only with regard to the appearance of the articular tissues but also providing quantitative measurements of several joint tissues. As a downside, MRI can be more expensive than the determination of biological fluids.

Many of the existing OA-related fluid biomarkers have grown from an understanding of joint tissue metabolism. They reflect joint tissue catabolism/anabolism [33,34] to the extent that many of the products released within the joint tissues from an altered metabolism can by themselves be representative of a pathologic process and/or stimulate a catabolic response. As OA is a disease affecting the joint tissues, the search for biomarkers has naturally centered on molecules such as those principally associated with cartilage, bone metabolism, and inflammation. In the athlete population, in which exercise/training requires excessive knee loading, these types of molecules could be used to gather information relevant to the efficacy and limits of exercise/training routines.

## 3. Fluid Biomarkers

In the cartilage matrix, collagen type II and aggrecan are the most abundant proteins [35,36]. Their synthesis degradation products have been the focus of OA biomarker studies for many years [37].

In regard to collagen metabolism, several biomarkers associated either to its synthesis, such as collagen type II propeptides (CPII and PIIANP), or to its degradation, the carboxy-terminal telopeptides of collagen types I (CTX-I) and II (CTX-II), N-terminal telopeptides of type I collagen (NTX-I), and a collagen type II epitope created by collagenases cleavage (C2C), have been studied. Aggrecan metabolism has also been monitored for its synthesis with the chondroitin sulfate epitope 846 (CS 846), which maintains the stability of the collagen II network. Aggrecan degradation markers were also studied and include chondroitin sulfate and keratan sulfate.

Another group of biomarkers, noncollagenous proteins found in the cartilage, were studied [38,39,40,41,42,43]. These include cartilage oligomeric matrix protein (COMP), which maintains the stability of the collagen II network, hyaluronic acid (HA), a high-molecular-weight glycosaminoglycan and a major component of the connective tissue, cartilage glycoprotein 39 (HC gp39), believed to play a role in the inflammation process and tissue remodeling, glycoprotein cartilage intermediate layer protein 2 (CILP2), reported to participate in cartilage scaffolding, and enzyme metalloproteases (MMP)-1, -3, -9, as well as a desintegrin and metalloprotease with thrombospondin motif (ADAMTS)4. Inflammatory factors have also been investigated as potential OA biomarkers. These include C-reactive protein (CRP), interleukins (IL)-1, -6, and -8, IL-1 receptor antagonist (IL-1Ra), soluble IL-6 receptor (sIL-6R), tumor necrosis factor-alpha (TNF-α), a chemokine promoting macrophage migration, chemokine C-C motif ligand 3 (CCL3), and melanoma inhibitory activity (MIA) [38,39,40,41,42,43]. Table 1 summarizes the serum/urine biochemical markers that have been reported for knee early OA and exercise/training in athletes.

### 3.1. Collagen Metabolism

Serum levels of the collagen synthesis markers CPII and PIIANP have been reported to decrease in OA [51,62], and the CPII decrease was also observed in situ in OA knee cartilage [81]. There is no report of the effect of exercise on PIIANP levels, but CPII levels were found unchanged in runners, soccer, rugby, and volleyball players [52,53,60].

The levels of most collagen degradation markers were affected in both early OA individuals and exercise/training in athletes. In early OA individuals, C2C and CTX-II levels were either increased [44,50,55,56,57,58] or unchanged [51]. Both serum and urinary NTX-I levels were positively associated with OA progression [61]. As for exercise/training, the level change varied according to the sport. The levels of urinary CTX-II and NTX-I of rugby and soccer players were significantly increased compared with non-athlete controls [60]; this was also reported in rowers and cross-country runners, but not in swimmers [59]. Moreover, the ratio of CTX-II/CPII was found significantly higher in soccer players than in non-athletes [60], suggesting that in soccer players, collagen type II degradation is relatively increased compared to its synthesis. In contrast, NTX-I, CTX-I, and CTX-II levels were decreased in cyclists [54]. In volleyball players, C2C and CTX-II levels were reported as unchanged [53,59], and C2C levels were unchanged as well in marathoners [52,53].

Together, these data suggest that collagen type II synthesis decreases in early OA, and type I degradation increases both in early OA and by exercise involving intense joint loading. Although collagen type II degradation products are found to increase in high-joint-impact sports, this is not yet resolved for early OA.

### 3.2. Aggrecan Metabolism

There are relatively few studies on the involvement of the marker of aggrecan synthesis, epitope CS846, in either early OA or exercise/training in athletes. In one study [44], serum levels of this epitope were unchanged between individuals having no OA and those with pre-radiographic (early) OA. However, another study [81] reported that the epitope CS846, as detected in situ in the knee cartilage, was decreased by 10% in the cartilage having early lesions. There is no report on the effect of exercise/training for this epitope.

In regard to other aggrecan products, both chondroitin and keratan sulfate levels were found increased in early OA [45,46], but unchanged in marathoners, soccer players, and uphill walkers [47,48,49]. However, one study reported increased levels of keratan sulfate in marathoners [47].

To date, an increase in aggrecan chondroitin and keratan sulfate levels appears to be associated mainly with early OA.

### 3.3. Other Proteins

Other proteins have also been evaluated as potential biomarkers for OA. HA and particularly COMP have been the focus of several studies. Their levels differ depending on the study and/or the sport involved. In OA, one study found COMP levels unchanged [44], but others increased [46,55,73,74], and one reported a negative association of serum COMP with the incidence of knee OA [56]. Interestingly, in a systematic review, Ren and Krawetz [82] suggested that the serum COMP level may not be a reliable diagnostic biomarker for early OA as such individuals had not yet developed significant cartilage damage. The authors reason that serum COMP levels may be dominated by the turnover of other types of cartilage in the body (e.g., costal cartilage) rather than the damage of the knee cartilage. Elevated levels of HA were reported in early OA [51,55,74,79], but unchanged in other studies [44,46].

COMP and HA levels have also been studied in exercise/training. In one study, their levels were monitored in healthy adults in response to an uphill walk [48]. The experimental group walked nonstop for 14 km (5.97° incline), while those from the control group (matched participants) walked on a horizontal pathway. Individuals from the experimental group had significantly higher serum COMP levels but decreased HA levels immediately after the uphill walk. Those changes did not last for more than 24 h after the activity. The authors interpreted the increased COMP as the susceptibility of the articular cartilage to the increasing load, and the change in HA being associated with its clearance due to the highly physical activity, instead of a change in cartilage structure.

The levels of HA did not vary in marathoners, soccer players, and walkers [47,75]. Increased levels of COMP were reported in marathoners and walkers [52,66,67,68,75], but remained unchanged in cyclists and volleyball players [53,54]. In soccer players [76,77], training induced fluctuations in the serum COMP levels during a competitive season. One could speculate that these increases were the result of a higher cartilage turnover, but this is debatable as there was a return to near-baseline values not long after the exercise. Moreover, it is unclear whether this process had a negative influence on overall joint health. In another study, moderate walking affected the serum COMP levels [78]. Hence, blood samples were drawn from active individuals immediately before, as well as at 0.5, 1.5, 3.5, and 5.5 h after a 30 min walk. Data showed that the serum COMP increased immediately and 5.5 h after the 30 min walk. They concluded that, in a healthy population, everyday activities such as a 30 min walk would most likely stimulate cartilage turnover rather than initiate cartilage degradation.

HC gp39 serum levels have been found increased [57] or unchanged [55,73] in early OA. Only one study reported 56% increased levels in the blood of marathoners taken before and after a race [80].

CILP2 has not been studied in early OA but is highly homologous to cartilage intermediate layer protein 1 (CILP-1), which was shown to increase in patients with early stage OA [83]. In the only study done with athletes [53], the levels of CILP2, in addition to those of other biomarkers including CPII, COMP, sC2C, urine CTX-II, and collagenase-generated peptide(s) of collagen type II (C2C-HUSA), were compared with the baseline and a two-year follow-up in 19 former volleyball players. Data showed that only CILP2 was significantly higher at 2 years.

The enzymes, ADAMTS4 and MMP-1, -3, and -9, have also been monitored, to some extent, in both early OA and athletes. MMP-1 levels did not change in either early OA [72] or marathoners [52], while ADAMTS4 increased in early OA [72]. MMP-3 and -9 levels increased in early OA [65,73] and in marathoners [52,70], and MMP-9 in cyclists [71]. In one study [52], 36 ultramarathoners participated in a race comprising 64 running days without any days of rest. Serums were collected within 4 days before the race and on days 15, 31, 47, and 58 of the 64-day race; COMP, MMP-1, MMP-3, MMP-9, C2C, and CPII levels were determined. COMP, MMP-3, and MMP-9 levels significantly increased. Moreover, increased MMP-3 levels, but not MMP-9, were associated with changes in COMP throughout the ultramarathon race. The authors hypothesized that MMP-3 might be involved in the degradation of COMP.

In summary, only MMP-3 and MMP-9 were reported as having increased levels in both early OA and marathoners. Other biomarkers, such as COMP, did not reach consensus for early OA and sports.

### 3.4. Inflammatory Molecules

OA is a disease in which inflammatory molecules play a role. Interestingly, such mediators were found at a higher level in early OA and decreased in advanced disease [84], suggesting their pertinence in early OA.

The chemokine CCL3 was proposed as a marker of early cartilage degeneration [63]. In this study, CCL3 reached a sensitivity of 70% with a specificity of 96% in differentiating pre-X-ray in knee OA patients (with changes diagnosed by MRI or arthroscopy) from controls. However, this marker awaits further validation. In the case of athletes, one study reported increased gene expression of CCL3 in blood from cyclists from resting to maximal intensity of exercise and after 15 min of recovery [64].

MIA is a protein produced predominantly by malignant melanoma cells and chondrocytes [85,86]. Its serum levels are elevated in patients with another arthritis disease, rheumatoid arthritis, and associated with joint destruction [87]. Although MIA has not been yet investigated in early OA, its levels are increased in marathoners [66]. In this study, levels of MIA and the inflammatory biomarkers CRP, IL-1β, IL1-Ra, IL-6, sIL-6R, and TNF-α, and COMP were compared in marathoners, nonrunning healthy subjects, rheumatoid arthritis and OA patients [66]. Data showed that baseline serum levels of TNF-α, sIL-6R, COMP, and MIA were significantly elevated in marathoners compared to healthy controls. Of note, the elevated baseline levels of COMP in marathoners were similar to those in OA, and the MIA levels were comparable to those in rheumatoid arthritis but higher than in OA. Compared to the baseline, serum levels of COMP, IL-1Ra, IL-6, and TNF-α were increased during the marathon, but only CRP and MIA were increased after 24 and 48 h [66]. Considering all the values of the healthy controls and the marathoners, correlations were found for COMP with sIL-6R, TNF-α, IL-1Ra, and soluble TNF receptor II (sTNFRII) [66]. This suggests an association of COMP with some aspects of the inflammatory response. Data from this study [66] also showed that between the elevated biomarkers before and immediately after the run, COMP levels significantly correlated only with IL-1Ra levels. This indicates that damages and metabolic changes occurred simultaneously in the joint, thus having a counter-regulation of the inflammatory response.

In addition to the above-mentioned work of inflammatory biomarkers, the levels of CRP, IL-6, and TNF-α were reported in other studies. Although these biomarkers were found unchanged in early OA individuals [55,63,65], changes were observed in athletes. For marathoners, one study reported unchanged levels of TNF-α [67], others showed increased levels not only of TNF-α but also of CRP and IL-6 [66,67,68,70]. A comparison of the effect of different running distances (a marathon of 42 km and an ultramarathon of 200 km) on plasma levels of COMP and CRP [68] showed that during the marathon, COMP levels increased 1.6-fold and returned to the pre-race level 2 days after. For ultramarathoners, although an increase of 1.9-fold was found, contrasting to the marathoners, this was maintained until the third day, and declined to the pre-race level on the sixth day. For its part, the CRP level was unchanged during the marathon but elevated by 3.4-fold the first day, returning to the pre-race level on the fourth day. Again, contrary to the marathoners, the CRP level increased 40-fold in ultramarathoners and was still increased on the sixth day. The authors concluded [68] that long-distance running may induce more impact stress, and the required time for recovery may vary according to the running distance. Cycling increased IL-6 [71], but resistance training decreased it [69].

Information on the levels of sIL-6R and IL-1Ra in early OA has not yet been reported. However, both levels increased in marathoners [66], and the IL-1Ra level remained unchanged in resistance trainers [69]. IL-8 levels increased in both early OA [63] and resistance trainers [69]. Finally, IL-1β levels in early OA were unchanged [63] or increased [58], but were unchanged in resistance trainers [69].

In summary, only the inflammatory marker IL-8 was similarly increased in individuals being classified as early OA and in marathoners. Others, including CRP, IL-1Ra, IL-6, MIA, sIL-6R, and TNF-α, depending on the study, were found to increase in particular in marathoners at baseline, during, and/or after a run.

### 3.5. Summary of Fluid Biochemical Markers

Many biochemical markers have been investigated, although very few, including collagen type I degradation products (CTX-I and NTX-I), MMP-3, MMP-9, and IL-8, seem to stand out as being regulated similarly in both early OA and high-impact sports. However, there were too few studies to be able to conclude on their specific biomarker potential. Differences were observed as well between high-impact and non-impact sports; data indicate that the major change in the fluid biomarkers related to the cartilage/articular degradation appears limited to athletes undergoing intensive and prolonged weight-bearing exercise.

### 3.6. Clinical Relevance of Fluid Biochemical Markers

The common fluid biochemical markers of early OA and of exercise/training in athletes are of high clinical relevance for many reasons. First, it has been reported that high serum levels of each of the biomarkers, CTX-I, NTX-I, MMP-3, MMP-9, and IL-8, could be associated with OA progression. Hence, the increased levels of the biomarkers CTX-I and NTX-I were found associated with progressive radiographic OA [88,89]. As well, synovial fluid levels of IL-8 also demonstrated a strong association with the severity of OA radiographic scores [90]. In addition, the serum levels of MMP-3 were shown to have predictive validity and responsiveness to the progression of knee OA. By using the knee OA patient cohort from clinical trials, the serum levels of MMP-3, MMP-9, or CTX-I have been found predictive of the effects of drug treatments reducing cartilage losses [91,92]. Moreover, data also showed that treatment with doxycycline, an agent that exhibits inhibitory activity of several MMPs, is also protective against progressive post-traumatic OA [93]. However, to our knowledge, there have been no studies in athletes that have shown an association between these biomarkers and the development or progression of OA, nor regarding a strong OA outcome (e.g., knee replacement).

## 4. Imaging Markers

In OA, clinical examination is used to diagnose articular injury and imaging techniques, such as radiography and MRI, generally serve as an adjunct evaluation. However, in the last two decades, MRI has become an important tool to assess OA. In contrast to radiography, MRI is a sensitive noninvasive tool for the detection of early joint tissue alterations as well as enabling a direct visualization and, importantly, quantification of the articular tissue structure. Methods to assess the knee structure by MRI include semiquantitative (scoring) and quantitative evaluations.

The MRI semiquantitative techniques most commonly used are the Whole Organ MRI Score (WORMS), Boston Leeds Osteoarthritis Knee Score (BLOKS), and MRI OsteoArthritis Knee Score (MOAKS) [94,95,96]. They all consider many features and regions of the knee, providing a global assessment of the articulation.

With the development of MR sequences and semi- and automatic quantitative determinations of knee tissues, it has become possible to quantify not only the cartilage volume, its loss, average thickness, and morphology [97,98], but also the alteration of the menisci, ligament, infrapatellar fat pad, bone curvature/shape, bone marrow lesions (BMLs), and joint effusion/swelling [99,100,101,102,103,104,105]. All these joint tissues have been found to be involved in the OA pathological process. Moreover, meniscal extrusion, early ligamentous degeneration, and effusion/synovitis, were shown to precede the onset of radiographic knee OA in addition to increasing the risk for the development of accelerated knee OA [65,106,107]. Indicative of the OA incidence are the infrapatellar fat pad and the bone curvature and shape [103,105,108,109,110,111].

Table 2 lists the MRI’s commonly investigated knee tissue alterations comparing those reported for early OA and athletes.

### 4.1. Early Knee Osteoarthritis

As mentioned above, the cartilage is a commonly used tissue to evaluate knee damages. The earliest cartilage structural alteration includes increased water content [127], probably related to collagen damage, changes in its content and arrangement, as well as a decreased glycosaminoglycan concentration and proteoglycan size [128,129]. The most widely utilized quantitative MRI sequences to evaluate such knee alterations are T1, T2, and T1ρ relaxations [122,123,124,125,130,131]. T1 relates more to the measurement of the proteoglycan content than to the collagen architecture, T2 is inversely correlated with collagen network organization and structure and directly associated with free water content, whereas T1ρ is independent of collagen orientation and is sensitive to only proteoglycan variations [132,133,134,135,136,137]. It is suggested that T1ρ is well suited to differentiate the cartilage structure of healthy subjects from early OA patients and appeared more sensitive than T2 relaxations times [138]. However, in addition to detecting cartilage alteration, T2-weighted images are also best suited for identifying early injuries of the muscles, tendons, and ligaments, and depicting bone marrow abnormalities [106,112,117,139].

In recent years, ultrashort echo time (UTE)-MRI sequences have become available for high-resolution imaging and quantitative assessment of many tissues of the knee, including cartilage, menisci, tendons, and ligaments [140,141,142,143,144]. It should be noted that the T2 and T1ρ sequences are sensitive to tissue orientation due to the magic angle effect. Consequently, the T2 and T1ρ values may increase drastically when the collagen fibers have a specific orientation, and the assessment could far exceed changes caused by cartilage degeneration. This has given rise to the development of novel T1ρ sequences providing incentive for the magic angle effect. These include UTE-MRI combined with magnetization transfers (UTE-MT) as well adiabatic T1ρ (UTE-Adiab T1ρ) sequences [143,145]. Studies showed that these sequences are reliable to quantify the macromolecular content relative to water content in the tissue, supporting their potential for effective detection of cartilage degeneration [146,147]. However, to our knowledge, there has been no report demonstrating the association of early changes in knee tissues seen with these sequences and OA incidence and/or progression or the effect of exercise/training.

### 4.2. Sports

The effect of exercise/training on structural knee tissues evaluated by MRI was found to vary depending on the sport and the study.

The T1ρ and T2 evaluations were used in comparing marathoners and age- and gender-matched controls [119]. This study followed 10 marathoners 2 weeks before, within 48 h after, and 10 to 12 weeks after the marathon. Although runners did not demonstrate any gross morphological MRI changes after the marathon, significantly higher T2 and T1ρ post-marathon values were observed in all cartilage areas, except the lateral compartment; T1ρ values remained high after 3 months of reduced activity. In contrast, Heckelman et al. [126] reported that MRI performed on the dominant knee of eight runners before, immediately after, and 24 h after running 3 and 10 miles showed that the T1ρ relaxation times significantly decreased immediately after running. For either distance, there were no differences between pre-exercise and recovery T1ρ values, but a significantly higher decrease following the 10-mile run compared with 3-mile run was observed [126]. Comparison of impact (basketball) and non-impact (swimming) sports showed that basketball players had greater T1ρ values in the radial zone of the central third weight-bearing region of their cartilage [115].

The effect of cycling, swimming, running, or power striding in healthy young adults has been reported [89]. Data showed that the total cartilage volume significantly decreased after 12 weeks of running and cycling exercise, while there were no changes in swimmers and power striding people. Mosher et al. [118] reported by using T2 that 30 minutes of running produced knee tissue deformation, with 84% of subjects demonstrating a decrease in mean thickness of the femoral and tibial cartilage. Similar findings have been reported for experienced runners after a one-hour training run [88]. This study showed a significant decrease for the whole cartilage volume; the femoral and the lateral tibial cartilage bodies having the greatest reductions in volume. Decreased cartilage volume was also observed in MRI scans taken at the baseline and after a two-year follow-up period of volleyball players over the age of 40 years [53]. As mentioned in Section 3 (Fluid Biomarkers), there were increased levels of CILP-2 in volleyball players [53]. Interestingly, those levels appeared related to the cartilage thickness loss in individuals with increased risk of developing knee OA [53].

Other knee tissues have been evaluated by MRI. Knee scans were performed on six recreational and two semiprofessional runners before and after a marathon [114]. Seven of these runners failed to demonstrate bone marrow oedema, periosteal stress reactions, or joint effusions. Effusions were not noticed in skiers [26] and marathoners in some studies [119,121] but were detected in others [120]. Interestingly, Horga et al. [148] reported, in novice runners, an improvement to damage of the subchondral bone of the tibial and femoral condyles following the marathon, but a worsening of the patella cartilage, although asymptomatic. The same group performed another study [116] with 30 asymptomatic novice marathoners who completed the study and were evaluated with MRI before and after a marathon. Data showed that no further lesions appeared at a follow-up and, for some participants, pre-existing BMLs and cartilage lesions (*n* = 5 and 3, respectively) remained unchanged immediately after the marathon and were reduced to their extent 6 months later. Signs of lesions reversibility were also found at 2 weeks post-marathon for 10 of 18 bone marrow oedema-like signals, and 3 of 21 cartilage lesions decreased. Another study in marathoners [119] revealed no significant changes in meniscal or ligament pathologies. Comparison of asymptomatic young elite swimmers with control individuals who did not practice impact sports on a regular basis [113] showed that 69% of the swimmers presented one or more imaging abnormalities: oedema of the pinfrapatellar fat pad (53.8%), prefemoral fat pad (19%), and BMLs (26.9%), as well as joint effusion (15.3%), while the meniscus, ligaments, and cartilage did not show any significant changes [113]. Skiing was reported not to alter infrapatellar fat pad signal intensity, but demonstrated an increase in abnormalities in the distal insertion of the patellar tendon [26].

### 4.3. Summary of Magnetic Resonance Imaging Markers

MRI appears to be very informative early during the disease process and could be used in addition to the fluid biomarkers. But for few articular tissues, there is obviously no overall consensus as to the impact of a joint-loading sport on the knee as well as between different sports. There seems to be a consensus, however, on the association of knee joint tissue of runners and cartilage loss. Moreover, in a few studies, marathoners showed no change or even an improvement in their BMLs.

### 4.4. Clinical Relevance of Magnetic Resonance Imaging Markers

The clinical relevance of MRI findings of a loss of cartilage in early OA as well as in runners is obvious as cartilage damage has been linked to a primary OA outcome, the knee replacement [149]. For the marathoners, the possible improvement of damaged subchondral bone may be a sign of joint protection. Indeed, BMLs have been reported to be a strong risk factor not only to predict cartilage loss, but also to predict knee replacement [149,150,151,152]. Moreover, the OA-modifying structural effect of drug treatments demonstrated by clinical trials was found associated with a reduction of BMLs [151,153,154].

## 5. Research Avenues

Datasets on biomarkers in OA and in its early stages are becoming more abundant. An avenue for investigating biomarkers is to assess them in a more comprehensive manner using larger datasets and state-of-the-art approaches, rather than the traditional “one marker—one disease”. Such a monodimensional study is a major limitation and does not fully exploit the complex patterns underlying the available data. This could be due in part to the methodology used, which often includes conventional statistics. Researchers are now considering using machine/deep learning approaches, which are based on algorithms designed to deal with the uncertainty and imprecision, typically found in datasets. By using such strategies, the combination of biomarkers and machine/deep learning, studies show a better performance in terms of misclassification and related metrics (e.g., sensitivity and specificity). As an example, by employing a decision tree algorithm for classifying early OA versus control, 16 proteins differing between the two groups were identified with a misclassification error rate of only 0.071 [155]. In another study [42], the authors developed a diagnostic algorithm using machine learning modeling for the detection of early OA and identified a combination of three plasma proteins with 73% sensitivity and 87% specificity. Machine learning methodologies are also becoming an important tool for measuring knee structural alteration and are gaining popularity in athletic training, performance, and injury prediction and prevention [156,157,158].

## 6. Conclusions

Although potential markers have been identified in forecasting knee alterations in early OA and in athletes practicing joint-loading sports, there is not yet a signature biomarker for each of these conditions, not mentioning common ones. Variability among the results of the studies has to be resolved before identifying a given molecule/factor or a combination as specific biomarkers. Other facets not yet well studied in biomarker predictors for either group are the comparison between male and female individuals and the diurnal variation.

A larger sample size, gender discrimination, validation, and controlled trials are urgently needed to make both fields move forward. Standardization and harmonization of the procedures also need more effort. For example, for early OA, there is a lack of a defined rationale for participant selection, as work on biomarkers has been often limited by the use of individuals at a more moderate stage of the disease instead of a genuinely early stage. Hence, very often, radiography evaluation is used to select early OA individuals. However, it is well known that this method lacks sensitivity to identify knee tissue modifications at their beginning, in addition to not directly assessing some knee tissue (e.g., cartilage, menisci). In athletes, the conflicting results may also reflect methodological heterogeneity and studies are often set up as pilots or exploratory with very low sample sizes. Well-designed, prospective studies are needed to clarify the inconsistency among findings.

Moreover, given that a single biomarker can have some intrinsic shortcomings, its use as the only endpoint is unlikely. For fluid biomarkers, limitations include, to name a few, no direct correlation with joint structural changes, no well-defined age-related changes, no gender discrimination, and unrecognized heterogeneous disease phenotypes [41]. There is increasing evidence that the use of a panel of fluid biomarkers and even more so in combination with MRI features will be more promising, as they provide complementary information not only for the disease status but also regarding the dynamic changes occurring during joint degeneration. Interestingly, in a study classifying OA knee patient progressors with machine learning tools and using a combination of fluid biomarkers, MRI features, and other individual parameters, the authors concluded that MRI variables contributed more to such discrimination than did other variables, even fluid biochemical markers [159].

Lastly, a larger dataset should also be employed that will enable adoption of more powerful analysis techniques such as those associated with machine/deep learning. The latter could evaluate and even interpret the complex relations between the features.

The above-described multidimensional approach would not only open novel perspectives, but would also enable these two fields (predicting OA at an early stage in individuals and in athletes) to move forward.

## Figures and Tables

**Table 1:** Fluid biochemical markers of knee early osteoarthritis and of exercise/training in athletes.

	EARLY OA	ATHLETE
**Aggrecan**		
Aggrecan epitope 846 (CS846)	→ [44]	NR
Chondroitin sulfate	↑ [45,46]	→ (M,S) [47], → (UW) [48]
Keratan sulfate	↑ [46]	→ (M,S) [47,49], ↑ (M) [47]
**Collagen degradation**		
C2C	↑ [44,50], → [51]	→ (M,VB) [52,53]
CTX-I	NR	↓ (Cy) [54]
CTX-II	↑ [55,56,57,58], → [51,58]	↑ (S,M,R,Rb) [59,60], ↓ (Cy) [54],→ (Sw,VB) [53,59]
NTX-I	↑ [61]	↑ (S,R,M,Rb) [59,60], ↓ (Cy) [54], → (Sw) [59]
**Collagen synthesis**		
CPII	↓ [51]	→ (M,S,Rb,VB) [52,53,60]
PIIANP	↓ [62]	NR
**Inflammatory molecules**		
CCL3	↑ [63]	↑ (Cy) [64]
CRP	→ [55,65]	↑ (M) [66,67,68]
IL-1ß	↑ [58], → [63]	→ (RT) [69]
IL-1Ra	NR	↑ (M) [66], → (RT) [69]
IL-6	→ [63,65]	↑ (M,Cy) [66,67,70,71], ↓ (RT) [69]
IL-8	↑ [63]	↑ (RT) [69]
MIA	NR	↑ (M) [66]
sIL-6R	NR	↑ (M) [66]
TNF-α	→ [63]	↑ (M) [66,70], → (M) [67]
**Non-collagenous proteins**		
ADAMTS-4	↑ [72]	NR
CILP2	NR	↑ (VB) [53]
COMP	↑ [46,55,73,74], → [44], ↓ [56]	↑ (M,S,UW,W) [48,52,66,67,68,75,76,77,78], → (Cy,VB) [53,54]
HA	↑ [51,55,74,79], → [44,46]	→ (M,S,W) [47,75], ↓ (UW) [48]
HC gp39	↑ [57], → [55,73]	↑ (M) [80]
MMP-1	→ [72]	→ (M) [52]
MMP-3	↑ [65]	↑ (M) [52,80]
MMP-9	↑ [73]	↑ (M) [52,70], ↑ (Cy) [71]

Biochemical markers increased (↑), decreased (↓), or unchanged (→) in serum or urine in the knee for early osteoarthritis (OA) and athletes performing high-joint-loading exercise/training. Cy, cycling; M, marathon—running; NR, not reported; R, rowing; Rb, rugby; RT, resistance training; S, soccer; Sw, swimming; VB, volleyball; W, walking; UW, uphill walking.

**Table 2:** Magnetic resonance imaging markers of early osteoarthritis and of exercise/training in athletes.

	EARLY OA	ATHLETE
Abnormalities of the tendon	NR	↑ (Sk) [26]
Bone marrow lesion/oedema	↑ [106,112]	↑ (Sw) [113], → (M) [114,115], ↓ (M) [116]
Cartilage volume/thickness	↓ [117]	→ (Sw) [89], ↓ (M,Cy,VB) [53,88,89,118]
Cartilage damage	↑ [106]	→ (Sw) [113]
Degenerative ligaments	↑ [107]	→ (Sw) [113], → (M) [119]
Infrapatellar fat pad signal alteration	↑ [107]	↑ (Sw) [113], → (Sk) [26]
Joint effusion/swelling	↑ [65,107]	↑ (Sw,M) [113,120] → (Sk) [26], → (M) [114,119,121]
Meniscal damage	↑ [106,107]	→ (M,Sw) [113,119]
T1/T1ρ/T2 relaxation times	↓ T1 [122], ↑ T1ρ [123], ↑ T2 [122,123,124,125]	↓ T1ρ (M) [126], ↑ T1ρ (M,BB) [115,119], ↑ T2 (M) [119]

Magnetic resonance imaging markers increased (↑), decreased (↓) or unchanged (→) in serum or urine in the knee for early osteoarthritis (OA) and athletes performing high-joint-loading exercise/training. BB, basketball; Cy, cycling; M, marathon—running; NR, not reported; Sk, skiing; Sw, swimming; T1, longitudinal relaxation time; T1ρ, longitudinal relaxation time rho; T2, transverse relation time; VB, volleyball.

## Data Availability

Not applicable.
